# An Assessment of Genetic Diversity and Drought Tolerance in Argan Tree (*Argania spinosa*) Populations: Potential for the Development of Improved Drought Tolerance

**DOI:** 10.3389/fpls.2017.00276

**Published:** 2017-03-02

**Authors:** Abdelghani Chakhchar, Matthew Haworth, Cherkaoui El Modafar, Marco Lauteri, Claudia Mattioni, Said Wahbi, Mauro Centritto

**Affiliations:** ^1^Laboratoire de Biotechnologie Valorisation et Protection des Agroressources, Faculté des Sciences et Techniques Guéliz, Université Cadi AyyadMarrakech, Morocco; ^2^Tree and Timber Institute, National Research Council – Istituto per la Valorizzazione del Legno e delle Specie ArboreeFlorence, Italy; ^3^Institute of Agro-Environmental and Forest Biology, National Research Council – Istituto di Biologia Agroambientale e ForestalePorano, Italy; ^4^Laboratoire de Biotechnologie et Physiologie Végétales, Faculté des Sciences Semlalia, Université Cadi AyyadMarrakech, Morocco

**Keywords:** water deficit, stomatal conductance, vapor pressure deficit, carbon isotope discrimination, argan oil, simple sequence repeat markers, population genetics

## Abstract

The argan tree (*Argania spinosa*) occurs in a restricted area of Southwestern Morocco characterized by low water availability and high evapotranspirative demand. Despite the adaptation of the argan tree to drought stress, the extent of the argan forest has declined markedly due to increased aridity, land use changes and the expansion of olive cultivation. The oil of the argan seed is used for cooking and as the basis for numerous cosmetics. The identification of argan tree varieties with enhanced drought tolerance may minimize the economic losses associated with the decline of the argan forest and constrain the spread of desertification. In this study we collected argan ecotypes from four contrasting habitats and grew them under identical controlled environment conditions to investigate their response to drought. Leaf gas exchange analysis indicated that the argan ecotypes showed a high degree of adaptation to drought stress, maintaining photosynthetic activity at low levels of foliar water content and co-ordinating photosynthesis, stomatal behavior and metabolism. The stomata of the argan trees were highly sensitive to increased leaf to air vapor pressure deficit, representing an adaptation to growth in an arid environment where potential evapotranspiration is high. However, despite originating in contrasting environments, the four argan ecotypes exhibited similar gas exchange characteristics under both fully irrigated and water deficit conditions. Population genetic analyses using microsatellite markers indicated a high degree of relatedness between the four ecotypes; indicative of both artificial selection and the transport of ecotypes between different provinces throughout centuries of management of the argan forest. The majority of genetic variation across the four populations (71%) was observed between individuals, suggesting that improvement of argan is possible. Phenotypic screening of physiological responses to drought may prove effective in identifying individuals and then developing varieties with enhanced drought tolerance to enable the maintenance of argan production as climate change results in more frequent and severe drought events in Northern Africa.

## Introduction

**FIGURE 1:**
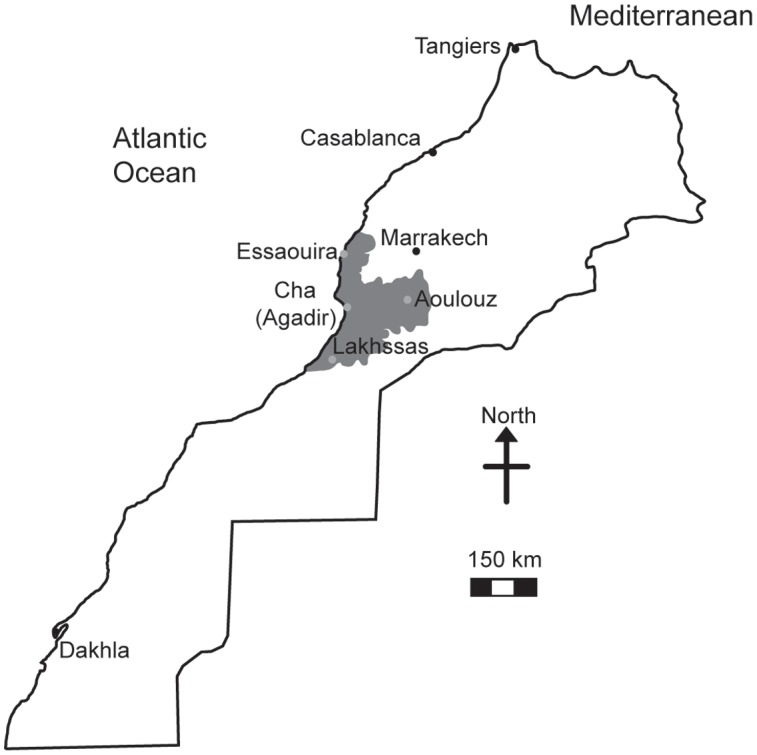
**Map of Morocco showing the distribution of the argan forest (dark gray shading) and the four locations where the argan ecotypes were collected (light gray circles)**.

The argan tree (*Argania spinosa*) is endemic to Southwestern Morocco (**Figure 1**) occupying a semi-arid to arid habitat (Lefhaili, 2010). The fruit of the argan tree is an important livestock feed and the oil produced by the seed has become increasingly valued for cosmetic purposes (Charrouf and Guillaume, 2008; Lybbert et al., 2010). Despite the social, agricultural and economic importance of the argan tree (Lybbert et al., 2002), the area of argan forest decreased 44.5% between 1970 and 2007 (de Waroux and Lambin, 2012) as part of a longer decline since the 18th century (McGregor et al., 2009). A major cause of this loss of argan forest has been attributed to increased aridity leading to desertification (de Waroux and Lambin, 2012; Alba-Sanchez et al., 2015) and the expansion of olive cultivation in the native argan forest (Charrouf and Guillaume, 2009). Nonetheless, the argan tree is highly adapted to growth in conditions characterized by drought and high temperatures (Diaz-Barradas et al., 2010) where mean annual precipitation ranges from 150 to 400 mm and temperatures can rise above 40°C (Bani-Aameur and Zahidi, 2005; Msanda et al., 2005). The argan tree has a highly effective water transport system to exploit the available soil moisture (Ain-Lhout et al., 2016) and during severe drought sheds leaves to reduce transpirative water-loss (Diaz-Barradas et al., 2010; Zahidi and Bani-Aameur, 2013). There are comparatively few studies that investigate the photosynthetic and stomatal responses of the argan tree to drought. Previous studies have indicated differences in the leaf water potential, antioxidant activity (Diaz-Barradas et al., 2010; Chakhchar et al., 2015, 2016), leaf morphology (Diaz Barradas et al., 2013; Chakhchar et al., 2015) and chlorophyll fluorescence parameters (Diaz-Barradas et al., 2010) response to drought of argan trees collected from different habitats. Despite the restricted range of the argan tree (950,000 ha: Lefhaili, 2010), genetic analyses have indicated variation between accessions collected from different habitats (hereafter referred to as ecotypes; El Bahloul et al., 2014) that may underpin this variety of response to water deficit. In this study we analyzed the gas exchange responses to water deficit of four argan ecotypes collected from contrasting habitats (**Figure 1** and **Table 1**). We hypothesize that the ecotypes from the most arid environments characterized by high evapotranspirative demand will exhibit the greatest tolerance to drought and enhanced water use efficiency (WUE). Alongside efforts to stabilize the argan forest through the creation of new plantations (Nouaim et al., 2002), analysis of physiological and genetic variability associated with increased drought tolerance may enable the identification of varieties that are more tolerant of increased aridity to prevent the further loss of argan forest area and to maintain the production of argan fruit, seeds and oil in its native habitat.

**Table 1:** A description of the sites where the argan ecotypes were collected (**Figure 1**).

Site	Annual rainfall (mm)	Monthly temperature (°C)			Average annual relative humidity (%)	Climate	Location	Altitude (m)	Soil
							
		Min.	Max.	Mean					
Essaouira	295	9.6	22.2	18.5	80–90	Cool temperate, semi-arid	31°52′72.20″N 9°76′83.33″W	181–226	Sandy-clay
Cha (Agadir)	225	7.2	27.1	20	75–85	Warm temperate, arid	30°35′16.81″N 9°47′69.78″W	275–430	Poorly drained sandy-clay containing stones and gravels
Aoulouz	232	5.6	35.7	21.6	60–70	Warm temperate, arid	30°68′82.38″N 8°15′86.00″W	700–850	Fertile clay
Lakhssas	189	7.3	31.2	20.9	50–60	Warm temperate, arid	29°57′66.22″N 9°71′27.15″W	916–988	Brown calcic containing stones and gravels


During episodes of drought, the level of water available in the soil for uptake by plants declines (Jones, 2007; Killi et al., 2016). As the root-zone soil dries, levels of free abscisic acid (ABA) in the leaves increase due to generation of ABA in the roots and transport in the xylem (Davies and Zhang, 1991), pH changes in the xylem sap (Wilkinson et al., 1998) and the conversion of glucose-conjugated ABA in the vacuole to free-ABA in the cytosol of leaf cells (Seiler et al., 2011). Higher [ABA] induces stomatal closure, reducing transpirative water-loss but also CO_2_-uptake for photosynthesis (*P*_N_; Tardieu and Davies, 1992; Sorrentino et al., 2016). As the concentration of CO_2_ in the sub-stomatal air-space (*C*_i_) falls, an increasing proportion of CO_2_ composed of the heavier carbon-13 isotope (^13^C) is taken up for *P*_N_. Exposure to water deficit over a sufficient period of time can result in a shift in the carbon isotopic composition of the leaf as tissues become enriched in ^13^C (Farquhar and Richards, 1984). Analysis of foliar carbon isotopic composition can therefore provide an indication of long-term WUE (Farquhar et al., 1989b).

Argan trees collected from coastal, inland and mountainous regions of Morocco exhibited differential responses to seasonal changes in temperature, relative humidity (RH) and water availability. Despite occupying the habitat with the greatest mean annual precipitation, the argan trees from the mountainous habitat exhibited the largest reduction in leaf water potential during the summer, corresponding to the lowest *P*_N_ values and quantum efficiency of CO_2_ assimilation of the three ecotypes (Diaz-Barradas et al., 2010). The argan trees growing under natural conditions showed close co-ordination of stomatal conductance (*G*_s_) with leaf to air vapor pressure deficit (VPD; Diaz Barradas et al., 2013) and leaf water potential (Diaz-Barradas et al., 2010). This suggests that the physiological and gas exchange responses of argan trees are highly adapted to growth in a habitat characterized by high evaporative demand and low water availability. The stomata of many plants close in response to a reduction in atmospheric humidity which induces an increase in leaf to air VPD (Mott and Peak, 2013). However, the stomatal response to increased leaf to air VPD depends upon the adaptation of a plant to the prevailing growth conditions, with plants grown under conditions of high evapotranspirative demand exhibiting greater stomatal sensitivity to VPD (Bauerle et al., 2004). Comparison of argan ecotypes under common garden conditions suggested that ecotypes from more arid habitats exhibited greater foliar levels of anti-oxidant activity and lower water potential values than their counterparts from regions with higher levels of rainfall (Chakhchar et al., 2015, 2016). It may therefore be expected that ecotypes from diverse habitats possess contrasting photosynthetic and stomatal responses to drought associated with underlying genetic variation.

Phenotypic screening of the physiological characteristics of plants combined with genetic sequencing can enable the identification and development of traits and/or varieties with desirable attributes such as high productivity or tolerance to abiotic stress (Flexas, 2016). We selected argan ecotypes from habitats with contrasting growth conditions (**Table 1**). These ecotypes were grown under controlled environmental conditions in a common garden experiment and exposed to water deficit. This study aimed to: (i) investigate the *P*_N_ and *G*_s_ responses of the argan tree to water deficit; (ii) gauge the stomatal adaptation of argan to growth in conditions of high evapotranspirative demand; (iii) characterize the WUE of the argan ecotypes to identify any traits/varieties that may confer improved tolerance to drought stress, and; (iv) assess genetic differences between the ecotypes that alongside their phenotypic responses to water deficit may be used to develop varieties of argan tree with enhanced tolerance to growth under water deficit conditions, and thus promote the stability of the native argan forest in Western Morocco (**Figure 1**).

## Materials and Methods

### Plant Material and Growth Conditions

Argan tree seedlings were collected from four localities: Lakhssas, a mountainous area of the Anti-Atlas mountains with the lowest mean annual precipitation of the sites; Cha is located at Agadir where the argan trees grow in a strongly maritime climate close to the Souss river, where the shallow water table provides high water availability for plant growth; Aoulouz is inland, upstream of the Souss valley, where RH is lower and water available for plant growth is lower than Cha (Agadir), and; Essaouira is the most northerly locality with an oceanic climate and highest annual rainfall. The location and description of the climate and soil types found at the sites are summarized in **Figure 1** and **Table 1**. One hundred seedlings were collected from trees used for agricultural production of argan fruit in each province in conjunction with The Regional Centre for Forest Research, Marrakech. Forty five seedlings of equal height were then selected for comparison of physiological responses to drought. The argan seedlings were approximately 15 cm high and grown in pots (4 L, 15 cm diameter) filled with a 45:45:10 mixture of soil, peat, and perlite in a common garden in Marrakech for 2 months prior to the experimental study. The argan tree ecotypes were transferred to a large walk-in growth chamber to compare their gas exchange responses under identical growth conditions. The growth chamber conditions were 400 μmol m^-2^ s^-1^ photosynthetically active radiation (PAR) for 16 h per day, a day/night temperature regime of 28/25°C and RH of 80%. After a period of 3 months to fully acclimate to the conditions in the growth chambers, water was withheld to half of the plants until the soil water content reached 25% of the full soil water holding capacity. Soil water holding capacity was determined by filling the pots with water and allowing them to free drain for 24 h before being weighed, this weight was assumed to represent the soil water holding capacity of the pots (Killi et al., 2014). Water in the pots was replenished every 2 days. Soil water was maintained at 100% of soil holding capacity in the control plants. This drought treatment was maintained for 2 months prior to the collection of gas exchange measurements.

### Leaf Gas Exchange and Analysis of Carbon Isotopic Composition

Measurement of leaf gas exchange was performed using a Li-Cor Li-6400 equipped with a 6400-05 conifer chamber (Li-Cor, Lincoln, NE, USA) between 08:00 and 12:00 each day. A metal halide light source was placed above the conifer cuvette at a height where PAR levels within the cuvette were 1000 μmol m^-2^ s^-1^. After each measurement, the area of the leaves within the cuvette was measured using a Li-Cor Li-3000 leaf area meter. Gas exchange parameters were then re-calculated using the corrected leaf area. Point measurements of *P*_N_, *G*_s_ and the internal sub-stomatal concentration of CO_2_ (*C*_i_) were taken on five replicate plants for each ecotype/treatment. Conditions within the cuvette were 1000 μmol m^-2^ s^-1^ PAR, 28°C and a RH of 60%. Instantaneous water use efficiency (WUE_i_) was calculated as the ratio of *P*_N_ to transpiration. The rate of respiration in the dark (*R*_N_) was determined by shutting off the light source and shading the leaves within the conifer cuvette for approximately 10 to 15 min until CO_2_ emission stabilized. This rate of CO_2_ emission was considered to represent *R*_N_ (Lauteri et al., 2014). To investigate the effect of leaf to air VPD on stomatal behavior further instantaneous measurements of *P*_N_ and *G*_s_ were conducted by reducing the RH of the air entering the cuvette (allowing a higher proportion of air to pass through the desiccant scrub tube) to increase leaf to air VPD. As stomata closed this resulted in an average 1.455 ± 0.0595°C increase in leaf temperature. After point measurements of gas exchange and measurement of leaf area, the leaves were destructively sampled and the relative water content of the leaves determined following Diaz-Pérez et al. (1995). To assess whether the argan ecotypes exhibit differences in photosynthetic capacity the response of *P*_N_ to increasing *C*_i_ was determined in the two ecotypes from latitudinal extremes (Essaouira and Lakhssas). To prevent any stomatal limitation to *P*_N_ the plants were exposed to a [CO_2_] level of 50 ppm for approximately 60 min to allow full stomatal opening. The level of [CO_2_] in the leaf cuvette was then rapidly increased in the following stages: 50, 100, 150, 200, 300, 500, 800, 1200, 1800, and 2200 ppm. Gas exchange parameters were recorded at each [CO_2_] level when *P*_N_ had remained stable for approximately 1 min (Centritto et al., 2003). The response of *P*_N_ to *C*_i_ was determined on five well-watered plants for each of the two argan ecotypes. The carboxylation capacity of ribulose-1,5-bisphosphate carboxylase/oxygenase (RubisCO; *V*c_max_), the maximum rate of electron transport required for ribulose-1,5-bisphosphate (RuBP) regeneration (*J*_max_) and the conductance of CO_2_ across the mesophyll (*G*_m_) were calculated following Ethier and Livingston (2004).

At the end of the experiment, leaf samples were collected for analysis of their stable carbon isotope composition. After collection, the leaves were dried at 60°C for 48 h until their weight remained stable. The leaves were then ground to a fine powder using a glass pestle and mortar. The ground leaf samples were then combusted in an elemental analyser (Model NA 1500, Carlo Erba, Milan, Italy) and the CO_2_ transferred in a helium flow to a continuous flow triple collector isotope ratio mass spectrometer (ISOPRIME, Manchester, UK). The isotope ratio ^13^C/^12^C was measured to calculate the samples’ carbon isotope composition (δ^13^C) relative to the VPDB (Vienna Pee Dee Belemnite) scale (Farquhar et al., 1989a).

### Analysis of Genomic DNA

Microsatellite markers are highly effective in population genetics studies due to their high polymorphism, co-dominance, multiallelism, abundance, and uniform dispersion in plant genomics (Gupta et al., 1996). Genomic DNA of the argan ecotypes was isolated by grinding 50–60 mg of fresh leaf tissue in a 2 mL microcentrifuge tube containing a 5 mm diameter steel ball. The ground tissue was cooled in liquid nitrogen and then homogenized using a Mixer Mill 300 (Qiagen, Hilden, Germany). Genomic DNA was then extracted and purified using the DNeasy96 Plant Kit (Qiagen). Four microsatellite primers (Mh04; Mh07; mVpCIRB03; mVpCIRB05) developed on *Manilkara huberi* and *Vitellaria paradoxa* (Azevedo et al., 2005; Cardi et al., 2005) of the Sapotaceae family alongside *A. spinosa* were used to determine the genetic diversity. The unbiased probability of identity (PIunb; Peakall and Smouse, 2006) was computed for the combination of the six markers was between 0.01 and 0.08. This value indicates the probability that two unrelated trees selected at random from a population would have identical genotypes at multiple loci: the lower this value, the higher the capacity of the markers used to capture the variability in the data set. Polymerase chain reactions were conducted using a GeneAmp 2700 Thermal Cycler (Applied Biosystems, Foster City, CA, USA). Twenty nanograms of genomic DNA was placed in 20 mL of reaction mix (Qiagen multiplex type-it kit) and exposed to the following cycles: 15 min at 95°C, 30 cycles for 30 s at 94°C, 90 s at 57°C, 1 min at 72°C and 30 min at 72°C. Amplification products (0.1 to 1 μL) were added to 20 μL formamide and 0.3 μL Genescan-500 ROX (Applied Biosystems, Foster City, CA, USA) and denatured at 95°C for 5 min. The samples were then run on an ABI PRISM 3100 DNA sequencer (Applied Biosystems).

Genotyper 3.7 software was used to score the alleles (Applied Biosciences). The programs Popgene 3.2 (Yeh et al., 1997) and GeneAlEx6 (Peakall and Smouse, 2006) were used to statistically assess intra and inter population genetic diversity. The total (N), observed (Na) and effective (Ne) number of alleles and then observed (Ho) and expected (He) heterozygosity were calculated. The Shannon Index (I) was calculated to characterize species diversity (Keylock, 2005) and unbiased heterozygosity (UHe) and the inbreeding co-efficient (Fis) were determined (Nei, 1978). The Nei genetic distance (Nei, 1978) and Unbiased Nei genetic distance (Nei and Roychoudhury, 1974) values were then used to generate unweighted pair group clustering (UPGMA: Unweighted Pair Group Method with Arithmetic Mean) using the software program POPTREE 2 (Takezaki et al., 2010). Analysis of molecular variance (AMOVA) was also performed to assess differences in population genetics between the argan ecotypes (Excoffier et al., 1992).

## Results

### Leaf Gas Exchange and Water Use Efficiency

**FIGURE 2:**
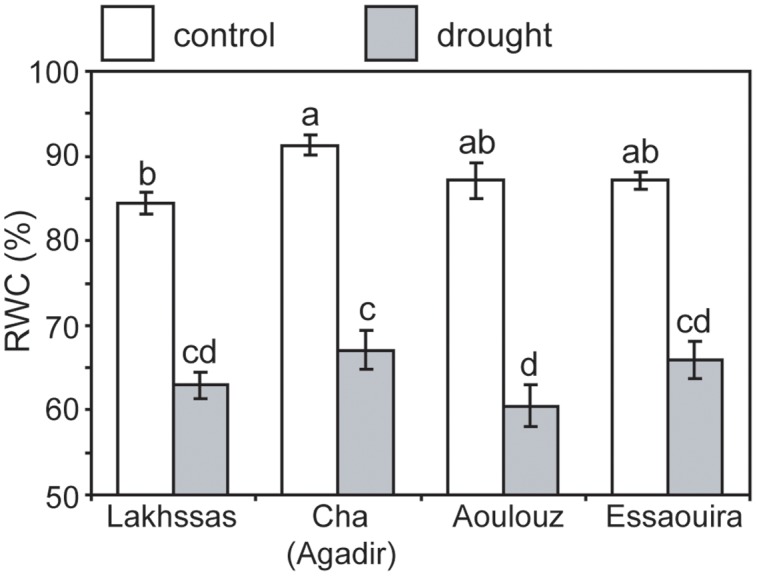
**Relative water content of argan leaves of the four ecotypes under control (white) and drought (gray) conditions.** Error bars indicate the standard error of five replicates. Letters denote homogenous groups using a one-way ANOVA and LSD *post hoc* test.

**FIGURE 3:**
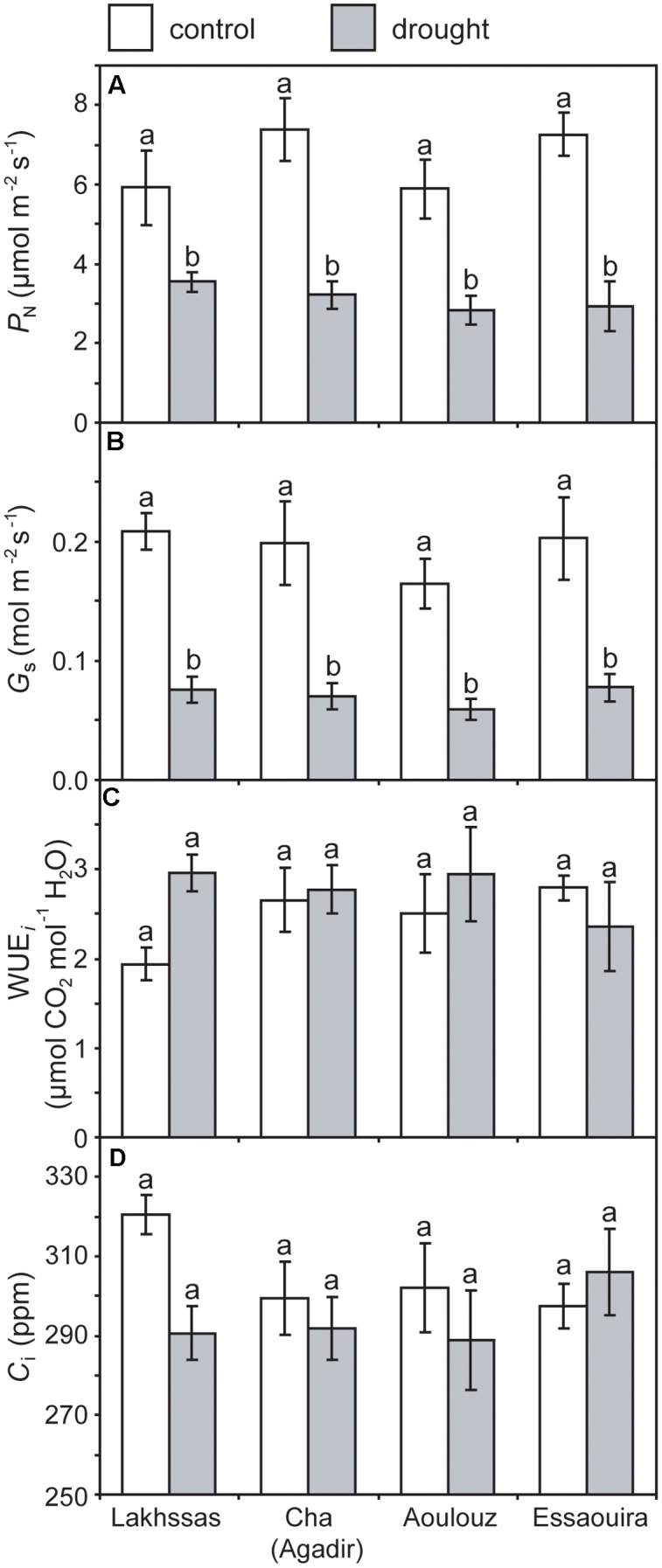
**Photosynthesis (*P*_N_)** **(A)**, stomatal conductance (*G*_s_) **(B)**, instantaneous water use efficiency (WUE_i_) **(C)** and internal sub-stomatal concentration of CO_2_ **(D)** of the four argan ecotypes under control (white) and drought (gray) conditions. Error bars indicate the standard error of five replicates. Letters denote homogenous groups using a one-way ANOVA and LSD *post hoc* test.

**FIGURE 4:**
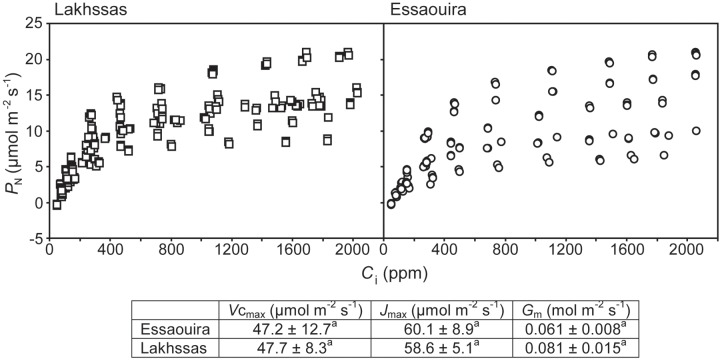
**The relationship between *P*_N_ and sub-stomatal [CO_2_] (*C*_i_) of the Lakhssas and Essaouira argan ecotypes from the latitudinal extremes of the range of the argan forest (**Figure 1**).** The carboxylation capacity of ribulose-1,5-bisphosphate carboxylase/oxygenase (RubisCO; *V*c_max_), the maximum rate of electron transport required for ribulose-1,5-bisphosphate (RuBP) regeneration (*J*_max_) and the conductance of CO_2_ across the mesophyll (*G*_m_) were calculated following Ethier and Livingston (2004). ± indicates one standard error. Letters denote homogenous groups using a one-way ANOVA and LSD *post hoc* test.

**FIGURE 5:**
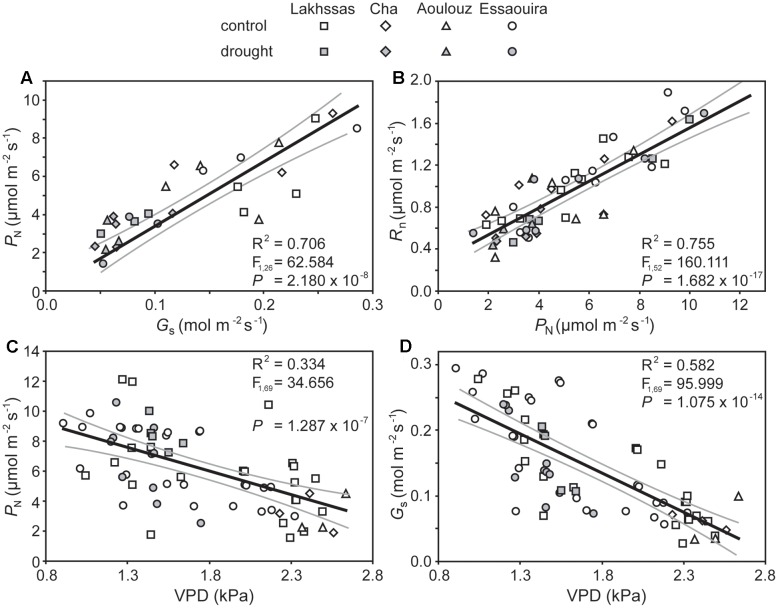
**Co-ordination of *P*_N_, *G*_s_ and respiration in the dark (*R*_N_) of the argan leaves in response to drought and increased leaf to air vapour pressure deficit (VPD):** **(A)** rates of *P*_N_ and *G*_s_; **(B)** the relationship between *R*_N_ and *P*_N_; **(C)** the effect of increased leaf to air VPD on *P*_N_, and; **(D)** the effect of increased leaf to air VPD on *G*_s_. Linear regression was performed to determine the significance of these relationships. The central black line indicates the line of best fit. The gray lines either side of the best fit line indicate 95% confidence intervals of the mean.

Growth in soils with water levels of 25% soil holding capacity induced reductions of 25 to 31% in the RWC of argan leaves; despite the moderate nature of the differences between ecotypes these were significant (**Figure 2**). The Aoulouz ecotype from the mountainous inland habitat showed the most pronounced reduction in RWC. The maritime influenced Essaouira and Lakhssas ecotypes from the latitudinal extremes of the argan forest (**Figure 1**) with the highest and lowest levels of precipitation both showed the lowest proportional reductions in RWC of ∼25% following water deficit (**Figure 2**). Under control and drought stress conditions the four argan ecotypes exhibited largely identical gas exchange characteristics (**Figure 3**). No statistical difference was observed in *P*_N_ (control, *P* = 0.556, *F*_3,15_ = 0.733; drought *P* = 0.773, *F*_3,15_ = 0.375), *G*_s_ (control, *P* = 0.711, *F*_3,15_ = 0.468; drought *P* = 0.770, *F*_3,15_ = 0.379), WUE_i_ (control, *P* = 0.293, *F*_3,15_ = 1.422; drought *P* = 0.704, *F*_3,15_ = 0.379) or *C*_i_ (control, *P* = 0.353, *F*_3,15_ = 1.220; drought *P* = 0.738, *F*_3,15_ = 0.428) between the four ecotypes under control or drought growth conditions. Water deficit reduced levels of *P*_N_ by 42–53% from 6–7.4 to 3.0–3.5 μmol m^-2^ s^-1^ (**Figure 3A**). This corresponded to a reduction in *G*_s_ values by 64–69% (**Figure 3B**). The reduction in levels of both *P*_N_ and *G*_s_ in plants grown in soils with lower water availability did not result in any change in WUE_i_ values (**Figure 3C**). This was consistent with no significant alteration in the δ^13^C values of the leaves of the argan ecotypes that experienced drought stress (**Table 2**). Analysis of the response of *P*_N_ to *C*_i_ in the Essaouira and Lakhssas ecotypes from the latitudinal extremes of the range of the argan tree indicated that there was no significant difference in underlying photosynthetic capacity between the two ecotypes (**Figure 4**), consistent with the similarity in *P*_N_ values recorded during point measurements of leaf gas exchange (**Figure 3A**). However, significant differences were found in the foliar δ^13^C values of the argan ecotypes under control conditions. The Essaouira ecotype from the habitat with the highest mean annual precipitation exhibited the most negative δ^13^C values, whilst the leaves of the Lakhssas ecotype exhibited the most positive δ^13^C values. However, a negative relationship between foliar δ^13^C and the mean annual precipitation of the source region of the ecotypes was not observed (linear regression, *F*_1,2_ = 5.910; *P* = 0.136). *P*_N_ rates of the argan trees were positively related to *G*_s_ (**Figure 5A**). Levels of *R*_n_ were also positively related to *P*_N_ (**Figure 5B**), indicative of co-ordination of metabolic and photosynthetic activity. Levels of both *P*_N_ (**Figure 5C**) and *G*_s_ (**Figure 5D**) declined with increasing leaf to air VPD, indicative of the modification of stomatal behavior to increasing potential evapotranspiration.

**Table 2:** Carbon isotope composition (δ^13^C) of leaf dry matter of the four argan ecotypes.

	δ^13^C of leaf material ‰			
	
	Lakhssas	Cha (Agadir)	Aoulouz	Essaouira
Control	-34.6 ± 1.1^ab^	-34.4 ± 0.7^ab^	-35.9 ± 0.6^bc^	-36.8 ± 0.4^c^
Drought	-34.5 ± 0.3^ab^	-34.0 ± 0.2^a^	-35.0 ± 0.6^ab^	-35.5 ± 0.5^abc^


*Values represent the mean of leaves from five replicate plants and ± indicates standard error. Letters indicate homogenous using a one-way ANOVA and LSD *post hoc* test.*

### Genetic Analyses

The four SSR markers produced reproducible amplifications that allowed the argan ecotypes to be distinguished. The mean number of alleles per locus was 2.85. Heterozygosis ranged from 0.00 (locus mVpCIRB03) to 0.65 (locus Mh07) with a mean of 0.305 per population (**Table 3**). For all loci, the He was larger than the observed (Ho) with the exception of Aoulouz, possibly indicative of non-random mating due to the presence of null alleles. At the population level, higher values of heterozygosity were observed for the Essaouira ecotype; however, this and the Agadir ecotypes showed significant and positive Fis consistent with a population inbreeding. All loci were polymorphic in the Essaouira ecotype, while the lowest level of polymorphism (50%) was observed in the Aoulouz ecotype (**Table 3**).

**Table 3:** Genetic diversity of four Argan populations: N, number of individuals, Ne, mean effective number of alleles per locus; Na, mean number of alleles per locus; PA, number of private alleles; Ho, observed heterozygosity; He, expected heterozygosity; I, Shannon index; UHe, unbiased heterozygosity, and; Fis, inbreeding coefficient.

population	N	Na	Ne	PA	I	Ho	He	UHe	Fis	Polymorphic Loci (%)
Essaouira	16.0	3.0 ± 1.0	2.194 ± 0.717	0.25 ± 0.25	0.745 ± 0.290	0.344 ± 0.131	0.419 ± 0.135	0.433 ± 0.140	0.325 ± 0.227	100
Lakhssas	20.0	2.8 ± 0.8	1.408 ± 0.213	0.25 ± 0.25	0.465 ± 0.188	0.225 ± 0.083	0.247 ± 0.096	0.254 ± 0.098	0.047 ± 0.072	75
Aoulouz	18.0	2.8 ± 1.1	1.604 ± 0.464	0.00 ± 0.00	0.476 ± 0.299	0.278 ± 0.165	0.249 ± 0.151	0.256 ± 0.155	-0.106 ± 0.044	50
Cha (Agadir)	16.0	2.8 ± 0.6	2.213 ± 0.533	0.50 ± 0.29	0.594 ± 0.268	0.281 ± 0.185	0.303 ± 0.151	0.313 ± 0.165	0.033 ± 0.197	75


*± indicates one standard error either side of the mean (*n* = 5).*

**FIGURE 6:**
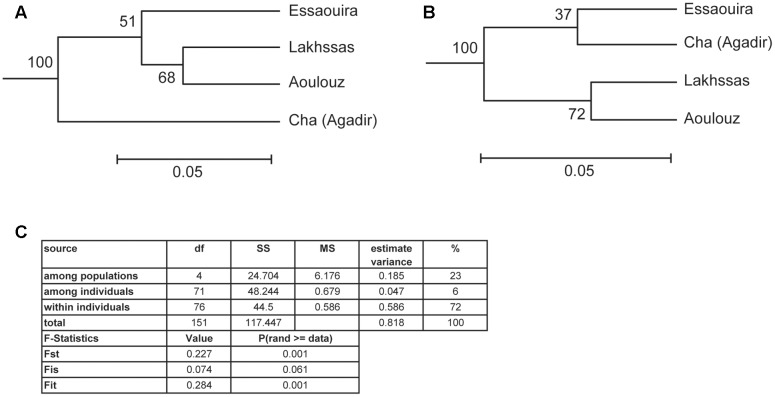
**Unweighted Pair Group Method with Arithmetic Mean (UPGMA) clustering analysis of the argan ecotypes based on:** **(A)** Nei biased, and **(B)** Nei unbiased. Scale indicates the genetic distance of Nei (1978) and suggests low genetic distance between the argan ecotypes collected from different provinces. **(C)** Analysis of molecular variance (AMOVA) analysis and F statistic of the argan ecotypes (Excoffier et al., 1992).

A higher number of effective alleles were observed in the Essaouira and Cha (Agadir) ecotypes in comparison to those derived from Lakhssas and Aoulouz (**Table 3**). The Cha (Agadir) ecotype showed the highest number of private alleles (PRA = 0.5), and alongside the Essaouira ecotype showed the highest level of genetic diversity (Cha, *I* = 0.594; Essaouira, *I* = 0.745). The lowest genetic variability was observed in the plants of the Lakhssas ecotype (*I* = 0.465). The Essaouira ecotype exhibited a significantly positive Fis-value of 0.325, indicative of inbreeding within the population. The UPGMA analysis indicated a low level of genetic divergence between the populations of ecotypes and no correspondences between the genetic and geographic distance (**Table 4**); the Nei biased (**Figure 6A**) and unbiased (**Figure 6B**) analyses produced contrasting results. The Nei biased analysis indicated that Essaouira and Cha (Agadir) were the two most divergent populations of ecotypes (**Figure 6A**) despite both occupying coastal habitats (**Figure 1**). The unbiased Nei analysis instead suggested that the coastal Essaouira and mountainous Aoulouz ecotypes were the most divergent (**Figure 6B**). The AMOVA analysis (**Figure 6C**) indicated 71% of variation occurred within the individuals analyzed, while the variation between different ecotype populations amounted to 23%.

**Table 4:** Pairwise population dissimilarity matrix of inbreeding co-efficient (Fis) values of the argan ecotypes.

	Essaouira	Lakhassas	Aoulouz	Cha (Agadir)
Essaouira	–	0.144	0.116	0.109
Lakhassas	0.144	–	0.128	0.321
Aoulouz	0.116	0.128	–	0.232
Cha (Agadir)	0.109	0.321	0.232	–


*This was then used to generate unweighted pair group clustering in **Figure 6**.*

## Discussion

The native argan forest of Western Morocco covers an area of 950,000 ha (Lefhaili, 2010). Despite this relatively restricted distribution, the argan forest occupies a range of diverse habitats ranging from cooler mountainous regions to plains where the climate is considerably warmer, and coastal to inland environments where RH and leaf to air VPD vary widely. It may therefore be expected to observe physiological and genetic variability between argan ecotypes adapted to these contrasting habitats; particularly, as under natural growth conditions argan trees exhibit differential photosynthetic and stomatal characteristics in this range of habitats (Diaz-Barradas et al., 2010). However, the results of this study showed little evidence to suggest physiological or genetic divergence between the populations of argan ecotypes analyzed; although a higher resolution of measurements may show differences in the progressive response of the argan ecotypes as soil dries.

### Leaf Gas Exchange Responses of Argan Ecotypes to Drought

Under drought stress argan dramatically modifies the level of osmolytes within leaves (Chakhchar et al., 2015). This may account for the maintenance of *P*_N_ and *G*_s_ to 3.5 μmol m^-2^ s^-1^ and 0.075 mol m^-2^ s^-1^ (**Figure 3**), respectively, when foliar RWC had fallen to ∼60% (**Figure 2**). Foliar water content is a strong controller of stomatal opening in many plants (Brown et al., 1976; Saliendra et al., 1995; Franks, 2013). In other drought adapted species such as olive (*Olea europaea*), a decline in RWC of 25 to 30%, equivalent to that observed in the argan ecotypes (**Figure 2**), resulted in a 94% reduction in *G*_s_ values and rates of respiration to exceed photosynthetic CO_2_ assimilation (i.e., negative *P*_N_ values; Sun et al., 2014). This indicates that argan possesses a high degree of osmoregulation, effective root systems for water uptake and also the capacity to maintain carboxylation and protective secondary metabolism during episodes of water deficit. The decline in *G*_s_ values following growth at 25% soil holding capacity for 2 months did not induce a significant reduction in *C*_i_ values (**Figure 3D**). Lower *C*_i_ under drought stress results in lower availability of CO_2_ for *P*_N_ (Flexas et al., 2002; Lauteri et al., 2014). The maintenance of *C*_i_ values under drought stress may be a function of lower demand associated with reduced carboxylation negating the impact of lower diffusion of on *C*_i_ values (accounting for the similarity in WUE_i_ values under control and drought stress conditions), the plants not closing stomata fully to maintain a degree of *G*_s_ (e.g., Haworth et al., 2015) or may reflect an adaptation of argan to drought stress that allows the retention of photosynthetic activity during episodes of reduced water availability (**Figure 3**). The lack of any significant alteration in the δ^13^C of the leaves of the argan ecotypes (**Table 2**) is likely associated with the constancy of *C*_i_ levels between control and drought treatments resulting in no change in discrimination of CO_2_ composed of the heavier ^13^C isotope (Farquhar et al., 1989b). The carbon isotopic measurements performed in this study involved analysis of the bulk leaf material. As such, the structural material within the leaf may reflect pre-stress growth conditions. Compound specific analysis of recently synthesized sugars may enable identification of the impact of drought stress on discrimination of carbon isotopes during CO_2_ assimilation in the argan ecotypes (e.g., Lauteri et al., 2014). The current dataset is comparatively variable, with a 2.4‰ range of δ^13^C values in control plants; this may constrain the effectiveness of bulk leaf carbon isotopes as a phenotyping tool in studies of argan and other sclerophylls such as olive (e.g., Sun et al., 2014). The soil water content of the pots was controlled every 2 days, this may have allowed the plants to utilize the proportion of water available for growth before water levels were replenished. The high frequency replacement of lost water to maintain a constant field capacity may not represent drought stress likely to occur under field conditions (Earl, 2003; Nemali and van Iersel, 2006). A lower field capacity or less frequent replenishment of soil water levels may result in the further stomatal closure and corresponding decline in *C*_i_ and *P*_N_ recorded in olive trees (Marino et al., 2014; Sun et al., 2014; Dbara et al., 2016).

The argan ecotypes showed identical reductions in *G*_s_ to reduce transpirative water-loss following growth under water deficit conditions (**Figure 3B**). *G*_s_ can be regulated via physiological regulation of stomatal aperture or modification of stomatal numbers in developing leaves (Haworth et al., 2015). Analysis of stomatal numbers in argan ecotypes collected from three contrasting regions indicated no population effect on stomatal density values (Bani-Aameur and Zahidi, 2005), suggesting that any ecotypic difference in stomatal control would be through active physiological behavior (e.g., Tomimatsu and Tang, 2012). The argan ecotypes analyzed in this study exhibited a high degree of active physiological stomatal behavior that allowed modification of *G*_s_ in response to changes in water availability (**Figure 3B**) and transportive demand (**Figure 5D**). The close co-ordination of *P*_N_ and *G*_s_ (**Figure 5A**) under control and water deficit conditions is consistent with other species adapted to growth in arid environments (e.g., Flexas et al., 2002; Marino et al., 2014; Sun et al., 2014). The link between *P*_N_ and the diffusive resistance to CO_2_ is also present during short-term variation in leaf to air VPD (**Figure 5C**) as the stomata close when evapotranspirative demand increases (**Figure 5D**). Over a range of leaf to air VPD of 0.8 to 2.8 the argan ecotypes exhibited a reduction of *G*_s_ values of 90%. In comparison, species from more mesic environments with higher water availability and lower potential evapotranspiration such as beech (*Fagus sylvatica*), chestnut (*Castanea sativa*) and oak (*Quercus robur*) showed respective *G*_s_ reductions of 33, 52, and 43% to an equivalent increase in leaf to air VPD (Heath, 1998). This stomatal sensitivity to VPD indicates that the argan tree possesses highly functional stomata. Moreover, the relationship between *P*_N_, *G*_s_ and *R*_N_ is indicative of a high level of co-ordination between mesophyll *P*_N_ and the regulation of stomatal aperture (Messinger et al., 2006; Hu et al., 2010; Engineer et al., 2016), as would be expected for a tree growing in an arid environment with high levels of evapotranspiration (Lefhaili, 2010) and risk of xylem embolism (Meinzer et al., 2009).

### Population Genetics of the Argan Ecotypes

Despite being collected from diverse habitats, the four argan ecotypes exhibited similar phenotypic responses to water deficit (**Figure 3** and **Table 2**). The four argan ecotypes exhibited similar photosynthetic and stomatal responses to growth in drought stressed conditions. This may suggest that the selective pressures experienced by the populations of argan ecotypes resulted in similar gas exchange responses as all of the habitats were characterized by comparatively low water availability (<300 mm per annum) and high evaporative demand (**Table 1**). Our results confirm the efficiency of using non-species-specific SSR markers in studies of genetic diversity. The possibility of using SSRs markers to perform cross-species amplification is an important tool to study the genetic characteristics of two or more species (Curtu et al., 2004; Sharma et al., 2009) Molecular ecologists increasingly require ‘universal’ genetic markers that can easily be transferred between species (Barbara et al., 2007). Moreover, despite the limited number of primers used the low value of PI confirms the reliability of the markers used in this study. Analysis of SSR markers in nine populations of argan trees suggested no difference in the number of observed (Ho) and expected (He) alleles (El Bahloul et al., 2014); indicative of the absence of evolutionary selective pressures influencing allele and genotype frequencies (Emigh, 1980). Previous analyses of isozymes (El Mousadik and Petit, 1996b), chloroplast DNA (El Mousadik and Petit, 1996a) and SSR markers (Majourhat et al., 2008) have suggested low diversity in the argan ecotypes studied. The analysis of SSR markers in this study would also be consistent with low genetic diversity among the argan ecotypes (**Figure 6** and **Table 4**). The population genetics of the argan ecotypes suggests a high degree of artificial selection (cf. El Bahloul et al., 2014), possibly associated with human management of the argan forest over 100s of years (Ruas et al., 2016). As the native argan forest occurs over a comparatively small area (Lefhaili, 2010) the population genetic analyses undertaken in this study would suggest that seedlings have been traded and moved between regions. Moreover, the increase in the intensity of grazing in recent years (Mellado, 1989; Alados and El Aich, 2008) may have prevented the establishment of smaller trees that have resulted from sexual reproduction; thus preventing the operation of drought induced selective pressures on any genetic variation resulting from sexual reproduction.

### Development of Increased Drought Tolerance in Argan

The native argan forest of Western morocco has been managed for hundreds of years (Ruas et al., 2011, 2016). This has resulted in argan populations from different areas being strongly related (**Tables 3**, **4**) (El Mousadik and Petit, 1996a,b; Majourhat et al., 2008). The argan ecotypes analyzed in this study showed a similar high degree of adaptation to drought stress in terms of gas exchange, metabolism, and photosynthetic activity (**Figures 3**–**5**). Nevertheless, this study has indicated that the vast majority of the variation within the argan ecotypes (71%) occurred within individuals (**Figure 6**). This raises the possibility that phenotypic screening (Kamoshita et al., 2008) and analysis of DNA/RNA (Deyholos, 2010) could be used to identify and develop traits/varieties that confer further drought tolerance. The use of rapid phenotyping techniques such as chlorophyll fluorescence and reflectance (Furbank and Tester, 2011; Fiorani and Schurr, 2013) would be highly effective in quickly assessing large numbers of individuals to identify those with favorable performance during water deficit for more in-depth gas exchange and molecular analysis. A series of common garden experiments at different locations within the range of the argan forest would permit selection of individuals suited to growth during water deficit in the specific conditions of mountains, coastal and inland habitats that make-up the argan forest. The identification of individual argan trees with enhanced drought tolerance could be an effective tool in minimizing the further loss of argan forest and preventing desertification.

## Author Contributions

AC, CEM, ML, CM, SW, and MC conducted the experiment. MH and MC wrote the manuscript.

## Conflict of Interest Statement

The authors declare that the research was conducted in the absence of any commercial or financial relationships that could be construed as a potential conflict of interest.
